# Grading diastolic function by echocardiography: hemodynamic validation of existing guidelines

**DOI:** 10.1186/s12947-015-0023-6

**Published:** 2015-06-24

**Authors:** Andrew D. M. Grant, Kazuaki Negishi, Tomoko Negishi, Patrick Collier, Samir R. Kapadia, James D. Thomas, Thomas H. Marwick, Brian P. Griffin, Zoran B. Popović

**Affiliations:** Libin Cardiovascular Institute, University of Calgary, Calgary, Canada; Menzies Research Institute, University of Tasmania, Tasmania, Australia; Heart and Vascular Institute, Cleveland Clinic, Cleveland, USA; Northwestern University Feinberg School of Medicine, Chicago, USA; Department of Cardiovascular Medicine, Cleveland Clinic, 9500 Euclid Avenue, Desk J1-5, Cleveland, OH 44195 USA

**Keywords:** Diastolic function, Diastolic dysfunction, Left ventricular end-diastolic pressure, Left ventricular filling pressure, E/A ratio, Tissue Doppler echocardiography

## Abstract

**Background:**

While echocardiographic grading of left ventricular (LV) diastolic dysfunction (DD) is used every day, the relationship between echocardiographic DD grade and hemodynamic abnormalities is uncertain.

**Methods:**

We identified 460 consecutive patients who underwent transthoracic echocardiography within 24 h of elective left heart catheterization and had: normal sinus rhythm, no confounding structural heart disease, no change in medications between catheterization and echo, and complete echocardiographic data. Patients were grouped based on echocardiographic DD grade. Hemodynamic tracings were used to determine time constant of isovolumic pressure decay (Tau), LV end-diastolic pressure (LVEDP) and end-diastolic volume index at a pressure of 20 mmHg (EDVi20).

**Results:**

Normal diastolic function was found in 55 (12.0 %) patients, while 132 (28.7 %) patients had grade 1, 156 (33.9 %) grade 2 and 117 (25.4 %) grade 3 DD. The median value for Tau was 46.9 ms for the overall population (interquartile range 38.6-58.1 ms), with a prevalence of a prolonged Tau (>48 ms) of 47.5 %. While there was an association between DD grade and Tau (p = 0.003), LV dysfunction (ejection fraction <50 %) was more strongly associated with increased Tau (p < 0.001) than was DD grade (p = 0.19). There was also an association between DD grade and LVEDP (p < 0.001), with both LV dysfunction (p = 0.029) and DD grade (p < 0.001) independently associated with LVEDP. Calculated EDVi20 was related to DD grade, but this relationship was driven by findings of paradoxically increased compliance in patients with severe DD.

**Conclusions:**

Although echocardiographic grading of DD was related to invasive hemodynamics in this population, the relationship was modest.

## Background

It is generally accepted that mild impairments of left ventricular diastolic function manifest as delayed early relaxation. The adequacy of the initial phase of diastole is reflected by Tau, the time constant of ventricular pressure decay [1, 2]. The pattern of echocardiographic findings described as grade 1 diastolic dysfunction (DD) is felt to represent an isolated early relaxation abnormality.

On the opposite end of the spectrum, severe impairments of diastolic function result in reduced chamber compliance with ‘restrictive’ ventricular filling. This is best characterized invasively using pressure-volume loop analysis. Due to technical, cost and safety issues, it is commonly assessed by measurement of left ventricular end-diastolic pressure (LVEDP). An alternative approach has been reported to estimate chamber compliance using extrapolation of end-diastolic volume index at a pressure of 20 mmHg [3, 4]. The echo pattern which denotes restrictive filling is referred to as grade 3 DD. An intermediate stage of DD (grade 2) is suggested to represent impaired relaxation, but with modestly elevated LVEDP [2].

The prognostic importance of the DD grading system has been shown in a variety of populations [5–9]. In keeping with the notion of a continuous process, an increase in DD severity over time has been documented in some individuals, and shown to be a determinant of symptoms [10] and mortality [11, 12]. Unfortunately, hemodynamic impairments in DD are non-uniform between subjects, and the relationship between hemodynamic and echo findings can be confounded [2, 13–18]. Patients with grade 1 DD can unexpectedly have elevated LVEDPs [15], while in patients with grade 2 DD, resting LV filling pressures may be elevated [16, 17], or within the normal range [18, 19]. Additionally, Tau prolongation is not a universal finding in patients with DD [15]. In light of these discrepancies, we sought to report invasive hemodynamics in a large set of patients with varying degrees of DD.

## Methods

### Subjects

Patients undergoing left heart catheterization and trans-thoracic echocardiography within 24 h were identified by retrospective review of echocardiography and catheterization databases at the Cleveland Clinic from Jan 2008 until Oct 2010. Patients with poor echo image quality (inability to adequately delineate left atrial or left ventricular borders) or incomplete mitral inflow or tissue Doppler data were excluded. Further exclusion criteria included atrial fibrillation, heart rate > 100 beats per minute, mitral stenosis or severe mitral annular calcification, severe mitral or aortic regurgitation, acute coronary syndrome, and prior heart transplantation. Manual review of electronic medical records was used to determine any change in diuretics or vasodilators between cardiac catheterization and echocardiography, which was also considered an exclusion criterion.

### Echocardiography

Trans-thoracic echocardiograms were completed according to laboratory protocol. Archived images were re-analyzed by three blinded investigators with re-measurement of all relevant parameters. These included left atrial volume measurements, peak early (E) and atrial (A) velocities of mitral inflow, A wave duration, early mitral inflow deceleration time (DT), septal and lateral mitral annular e’ velocities, pulmonary vein A wave reversal duration, isovolumic relaxation time (IVRT), and time difference between the onset of the E wave and e’. Where possible, each measurement was averaged over multiple cardiac cycles.

**Fig. 1:**
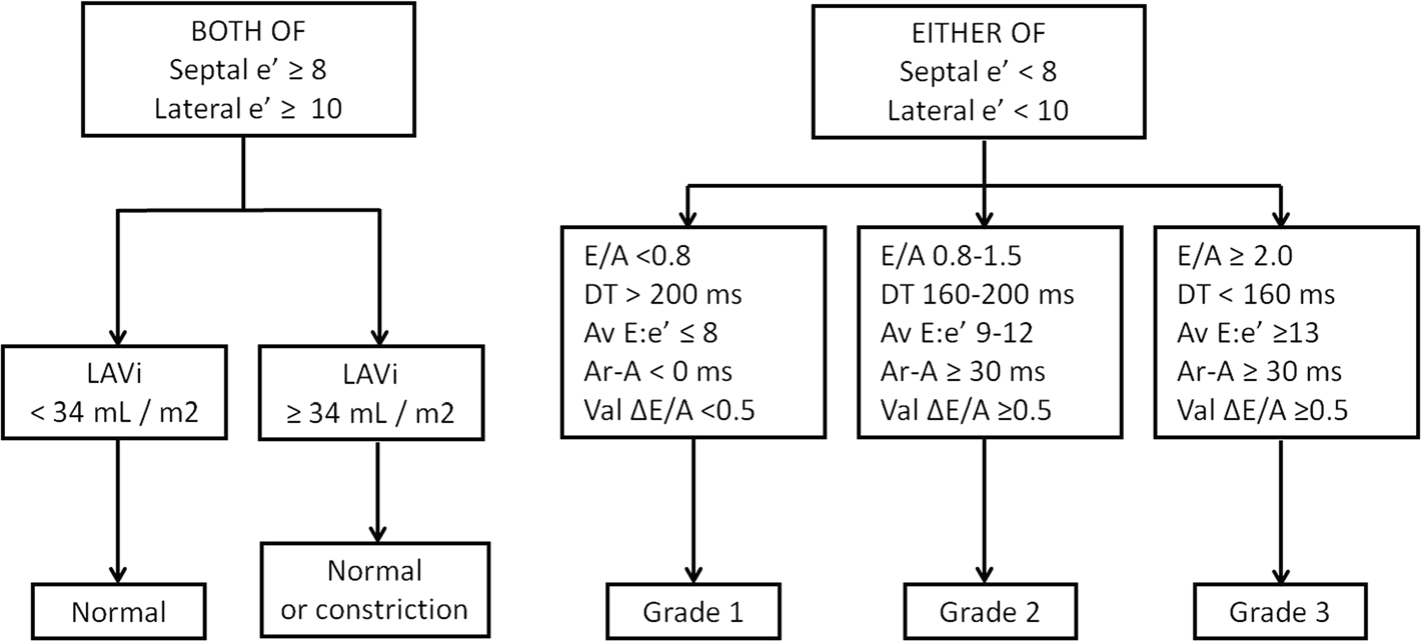
Implementation of guideline recommendations for diastolic function grading modified from reference 2. Abbreviations: DT = deceleration time; A = duration of atrial wave on mitral inflow; Ar = duration of pulmonary vein flow A wave reversal; Av E/e’: ratio between peak early velocity of mitral inflow and the average of early septal and early lateral mitral annulus peak velocities; E/A: ratio between peak velocities of early and late (atrial) waves seen on of mitral inflow; Val Δ E/A = change with Valsalva of E/A ratio

Diastolic function grading was assigned based on current guidelines [2] (see Fig. 1). In cases where parameters were non-congruent, DD grade was established as that with the highest number of characteristic parameters assuming equal weighting.

### Cardiac catheterization

Subjects underwent left heart catheterization by a standard approach in a fasting state. All measurements were made using fluid-filled catheters. Left ventricular end-diastolic pressure (LVEDP) was determined post-A wave. The time constant of isovolumic pressure decay (Tau) was estimated with a zero asymptote assumption as follows [20]:

$$ Tau = IVRT\ /\ \left[Ln\left({P}_{systolic}\right)\ \hbox{--}\ Ln\ (LVEDP)\right] $$

Where IVRT is isovolumic relaxation time by Doppler echocardiography, P_systolic_ peak LV systolic pressure and LVEDP LV end-diastolic pressure. Projected end-diastolic volume index at a pressure of 20 mmHg (EDVi20) was calculated as previously reported [3, 4].

### Statistics

Patients were grouped according to echocardiographic diastolic function grade. Categorical variables were compared between groups with chi-squared analysis. Continuous variables were compared between groups using analysis of variance (ANOVA) or Kruskal-Wallis where appropriate.

The influence of DD grade on each of Tau, LVEDP and EDVi20 was tested in the entire population, and then separately in those with impaired (LVEF <0.50) and preserved (LVEF ≥ 0.50) left ventricular systolic function. The same relationships were then assessed with multivariable analysis using DD grade and LV dysfunction (again defined as LVEF <0.50) as independent factors. Statistical analyses were repeated after excluding all patients with abnormal septal or lateral e’ velocity but left atrial volume index <34 mL/m^2^.

A p value of <0.05 was considered statistically significant. Analyses were performed using SPSS Statistics 19.0 (IBM Corp. Armonk, NY).

## Results

### Subjects

**Fig. 2:**
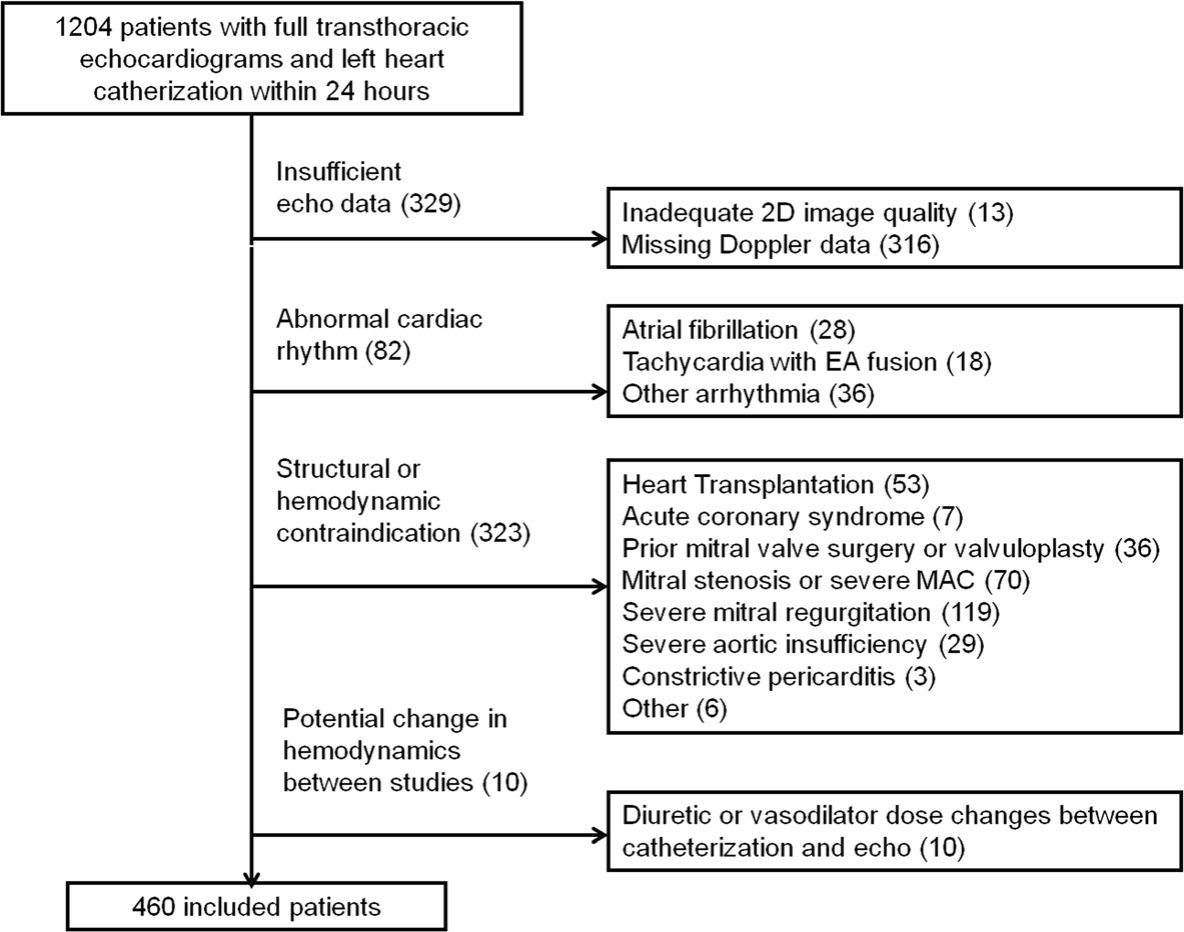
Flow chart of patient selection based on imaging and clinical criteria

From 2008 to 2010, a total of 1204 patients underwent an elective transthoracic echocardiogram within 24 h of left heart catheterization at our institution. Thirteen studies were excluded for poor image quality and 316 due to incomplete Doppler or other data. A further 415 patients were excluded for clinical reasons (see Fig. 2).

**Fig. 3:**
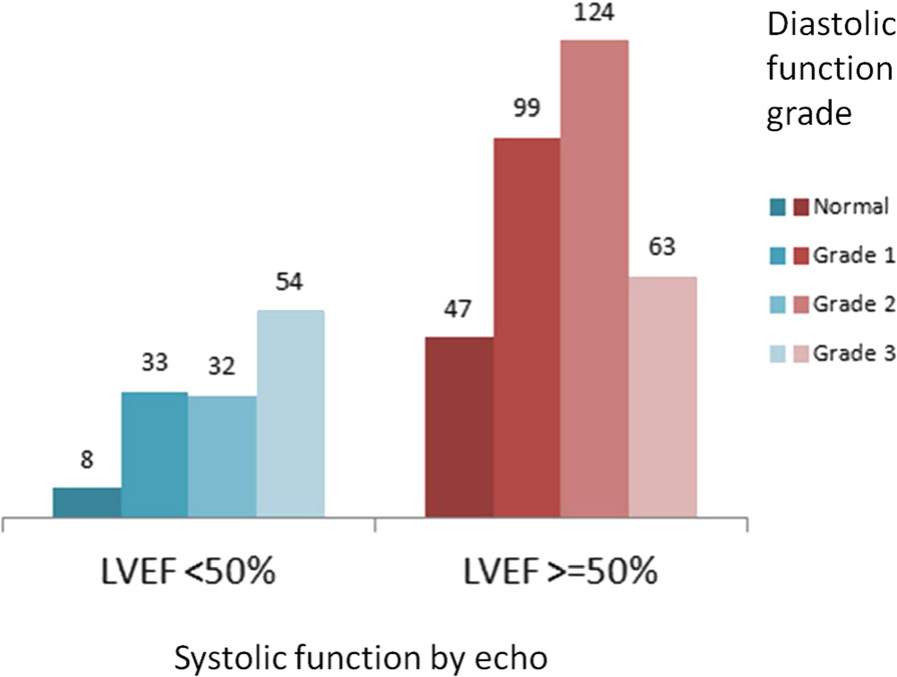
Number of patients with each diastolic function grade according to left ventricular ejection fraction (LVEF)

Among the remaining 460 patients, 55 (12.0 %) had normal diastolic function, 132 (28.7 %) had grade 1, 156 (33.9 %) grade 2 and 117 (25.4 %) grade 3 DD (Fig. 3). Demographics and clinical characteristics of are reported in Table 1.

**Table 1:** Patient Demographics, echocardiographic parameters and Invasive Hemodynamics

	Normal	Grade 1	Grade 2	Grade 3	P value
N	55	132	156	117	-
Age	51.4 ± 13.1	66.0 ± 12.0	62.7 ± 11.5	64.8 ± 14.4	<0.001
Male Gender	35/55 (63.6 %)	85/132 (64.4 %)	95/156 (60.9 %)	66/117 (56.4 %)	0.54
White Race	42/53 (79.2 %)	105/130 (80.8 %)	122/153 (79.7 %)	89/115 (77.4 %)	0.93
Cath indication					<0.001
CAD	45 (81.8 %)	62 (47.0 %)	103 (66.0 %)	37 (31.6%)	
Aortic stenosis	0 (0.0 %)	21 (15.9 %)	12 (7.7 %)	20 (17.1 %)	
LV dysfunction	6 (10.9 %)	28 (21.2 %)	26 (16.7 %)	38 (32.5%)	
LV hypertrophy	3 (5.5 %)	21 (15.9 %)	13 (8.3 %)	18 (15.4%)	
Other	1 (1.8 %)	0 (0.0 %)	2 (1.3 %)	4 (3.4 %)	
BMI	28.4 (23.1-32.3)	28.8 (25.2-31.2)	27.3 (24.6-31.2)	28.9 (25.3-32.9)	0.39
BNP	34 (18-117)	77 (30-180)	108 (29-298)	210 (83-556)	<0.001
LVEF	58 % (53-64 %)	60 % (50-66 %)	57 % (51-63 %)	51 % (38-60 %)	<0.001
LA volume index	23.7 (18.4-30.7)	26.1 (18.7-34.2)	26.5 (21.2-36.2)	35.4 (25.3-47.4)	<0.001
E/A ratio	1.5 (1.2-1.8)	0.70 (0.61-0.79)	1.0 (0.8-1.2)	1.2 (0.9-2.0)	0.011
E/e’ ratio	7.0 (6.3-8.8)	8.3 (6.8-11.5)	10.4 (9.2-11.9)	15.7 (12.7-20.5)	<0.001
DT	182 (161-209)	250 (216-296)	190 (174-220)	160 (145-207)	<0.001
Systolic BP	127 (119-146)	139 (126-159)	140 (124-156)	138 (119-156)	0.012
Tau	43.8 (35.7-53.5)	51.3 (41.5-60.2)	44.8 (37.6-54.4)	47.5 (38.9-59.6)	0.003
LVEDP	16.0 (10.0-20.0)	14.0 (11.0-18.0)	14.0 (10.0-18.0)	18.0 (12.0-24.0)	<0.001

Data are presented as mean ± standard deviation where normally distributed or median (25^th^ to 75^th^ percentile). P values are for ANOVA across diastolic function grades

*BMI* body mass index, *BNP* B-type natriuretic peptide, *LVEF* left ventricular ejection fraction, *LA* left atrium, *DT* deceleration time, *IVRT* isovolumic relaxation time, *SBP* systolic blood pressure, *DBP* diastolic blood pressure, *LVEDP* left ventricular end-diastolic pressure, *CAD* coronary artery disease

### Tau and echocardiographic diastolic function

**Fig. 4:**
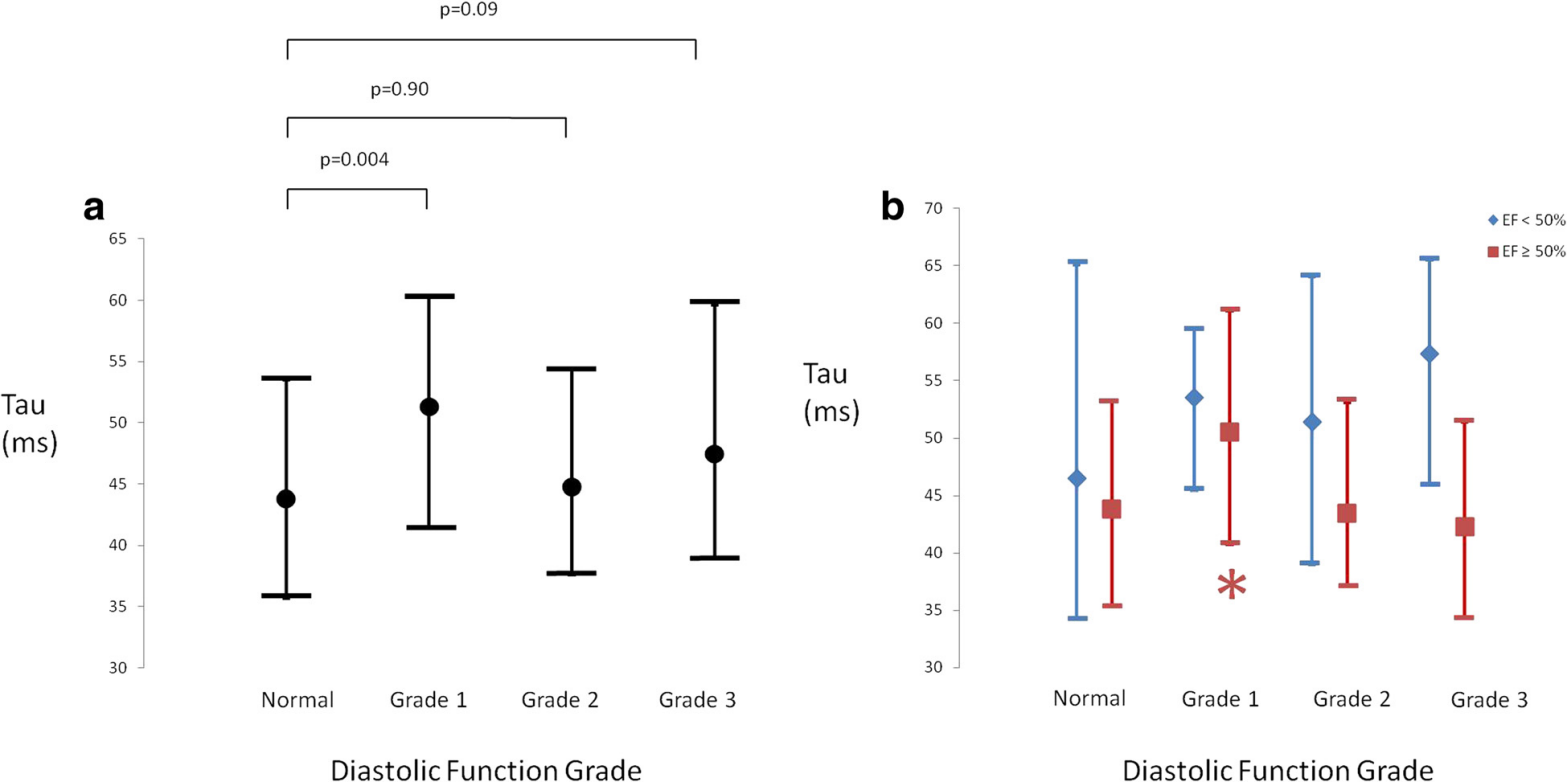
**a**. Time constant of isovolumic pressure decay (Tau) in patients stratified according to diastolic function grade. Data are shown as median with error bars representing 25 % and 75 % percentile. Patients with normal diastolic function had better relaxation (lower tau) than patients with Grade 1 diastolic dysfunction, but there were no differences between normal diastolic function and grade 2 or 3 diastolic dysfunction. **b**. Time constant of isovolumic pressure decay (Tau) in patients stratified according to diastolic function grade and presence or absence of left ventricular systolic dysfunction (EF < 50 %). Data are shown as median with error bars representing 25 % and 75 % percentile. *: p < 0.05 for comparison between patients with EF ≥ 50 % and Grade 1 diastolic dysfunction versus all other diastolic function subgroups with EF ≥ 50 %

The median value for Tau was 46.9 ms (interquartile range 38.6-58.1 ms), with a prevalence of a prolonged Tau (>48 ms) of 47.5 %. There was an association between DD grade and Tau by Kruskal-Wallis analysis (p = 0.003) as depicted in Table 1 and Fig. 4a. Post-hoc analysis showed a difference between patients with normal diastolic function and those with grade 1 DD (p = 0.004). Grade 1 patients also differed from grade 2 (p = 0.02), but no differences were seen in the other between-group comparisons. The positive predictive value of DD of any grade for detecting Tau > 48 ms was 49.0%. Similar results were seen when patients with low septal or lateral e’ but left atrial volume index <34 mL/m^2^ were excluded (p = 0.023 for ANOVA, only significant difference between normals and grade 1, p = 0.031). In multivariable analysis, LV dysfunction was associated with increased Tau (p < 0.001, partial η^2^ = 0.032), but DD grade was not (p = 0.19, partial η^2^ = 0.012). Post hoc analysis of patients without LV dysfunction showed patients with Grade 1 DD to have higher Tau than any other subgroup (p < 0.05 for all comparisons). In contrast, there was no difference between DD subgroups in patients with LV dysfunction (Fig. 4b).

### Filling pressures and echocardiographic diastolic function

**Fig. 5:**
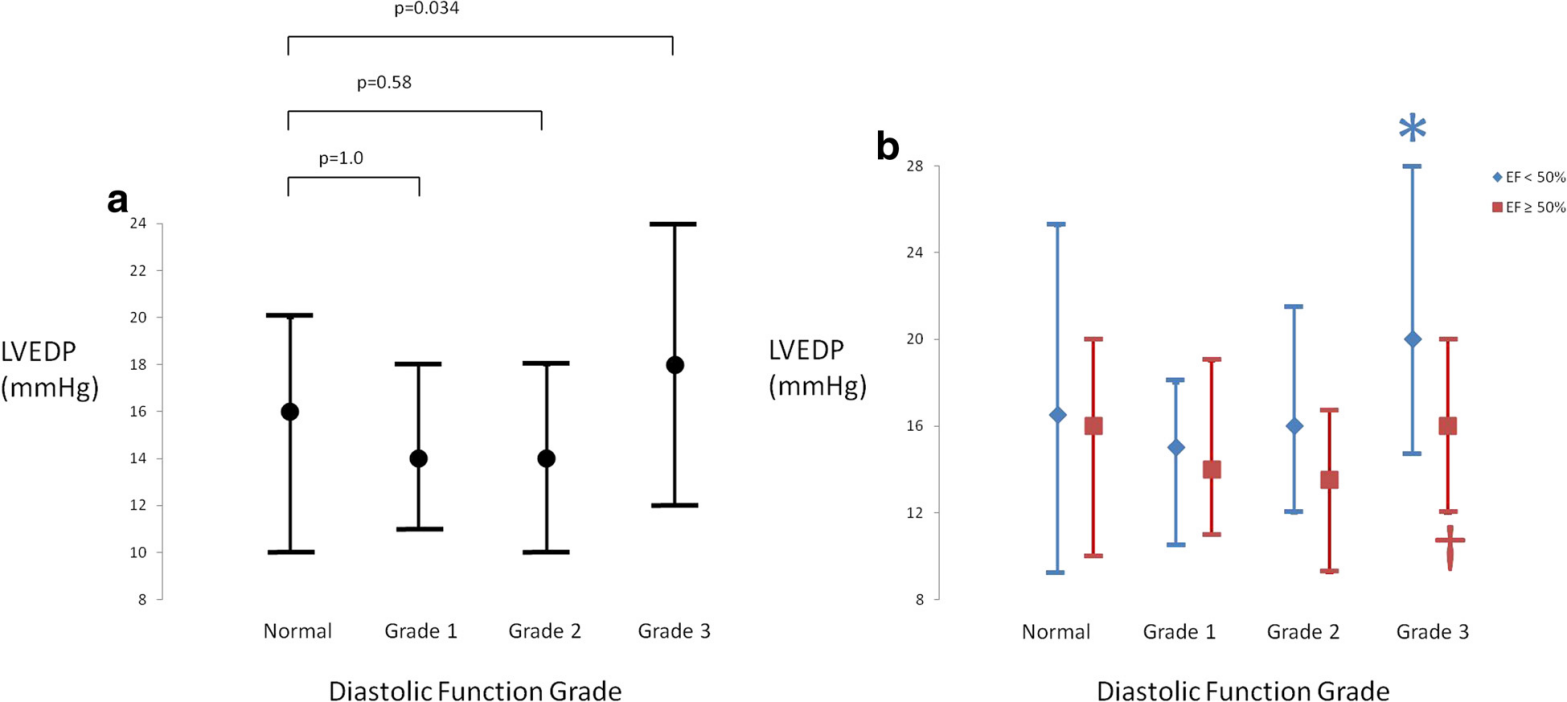
**a**. Left ventricular end-diastolic pressure (LVEDP) in patients stratified according to diastolic function grade. Data are shown as median with error bars representing 25 % and 75 % percentile. Patients with normal diastolic function had lower LVEDP than patients with Grade 3 diastolic dysfunction, but there were no differences between patients with normal diastolic function and grade 1 of 2 diastolic dysfunction. **b**. Left ventricular end-diastolic pressure (LVEDP) in patients stratified according to diastolic function grade and presence or absence of left ventricular systolic dysfunction (EF < 50 %). Data are shown as median with error bars representing 25 % and 75% percentile. *: p < 0.001 for the difference between grade 3 and grade 1 in patients with LVEF < 50 %; †: p = 0.02 for the difference between grade 3 and grade 2 In patients with LVEF ≥ 50 %. All other p values for post-hoc comparisons are >0.05

The median LVEDP was 15 mmHg (interquartile range 10-20 mmHg). There was an association between DD grade and LVEDP by Kruskal-Wallis (p < 0.001). Post-hoc analysis showed a difference in LVEDP between grade 3 DD patients and those with normal diastolic function (p = 0.034), grade 1 DD (p = 0.003) and grade 2 DD (p < 0.001) (Fig. 5a). When the analysis was repeated excluding patients with reduced septal or lateral e’ but left atrial volume index <34 mL/m^2^, there was no association between DD grade and LVEDP (p = 0.089). In multivariable analysis, LV dysfunction (p = 0.029, partial η^2^ = 0.011) and DD grade (p < 0.001, partial η^2^ = 0.049) were independently associated with LVEDP (Fig. 5b).

### End diastolic volume index at 20 mmHg and echocardiographic diastolic function

**Fig. 6:**
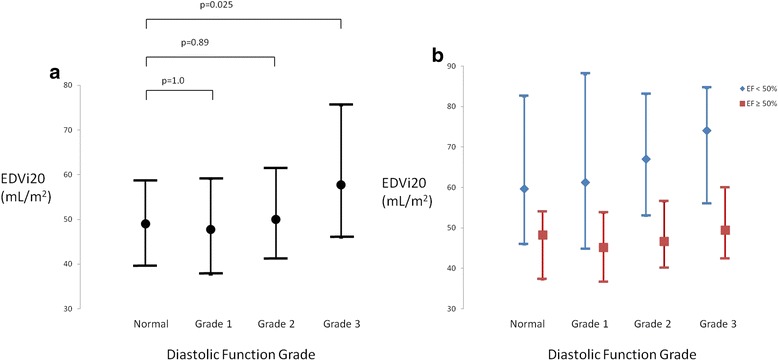
**a**. End-diastolic volume index at 20 mmHg (EDVi20) in patients stratified according to diastolic function grade. Data are shown as median with error bars representing 25 % and 75 % percentile. Patients with normal diastolic function had lower EDVi20 than patients with Grade 3 diastolic dysfunction, but there were no differences between patients with normal diastolic function and grade 1 of 2 diastolic dysfunction. **b**. End-diastolic volume index at 20 mmHg (EDVi20) in patients stratified according to diastolic function grade and presence or absence of left ventricular systolic dysfunction (EF < 50 %). Data are shown as median with error bars representing 25 % and 75 % percentile. No significant differences are seen between groups

The median value for EDVi20 was 47.5 mL/m^2^ (interquartile range 39.0 to 55.8 mL/m^2^). There was an association between DD grade and EDVi20 by ANOVA (p = 0.001). This was driven by grade 3 DD patients representing a distinct group (Fig. 6a), with EDVi20 significantly greater than in normal patients (p = 0.025), grade 1 (p = 0.001) and grade 2 (p = 0.022). When patients with low septal or lateral e’ values but left atrial volume index <34 mL/m^2^ were excluded, an association was still seen (p = 0.001, both grade 2 (p = 0.010) and grade 3 DD (p = 0.004) groups greater than normals). Multivariate analysis showed LV dysfunction (p < 0.001, partial η^2^ = 0.097) but not DD grade (p = 0.11, partial η^2^ = 0.013) to be associated with EDVi20. No subgroup differences were found when patients were broken down by LV function.

### Natriuretic peptides and echocardiographic diastolic function

An association was seen between B-type natriuretic peptide levels and DD grade (p < 0.001). On post-hoc analysis, the only distinct group was that of patients with grade 3 DD. This group differed from patients with normal diastolic function (p < 0.001), grade 1 DD (p < 0.001), and grade 2 DD (p = 0.006).

## Discussion

This study was an observational comparison of echocardiographic grading of diastolic dysfunction (DD) as recommended by major echocardiography societies [2, 21], with invasively derived hemodynamic data in patients who underwent echo and heart catheterization within 24 h. The major findings were that there was only a modest relationship between DD grade and invasively determined early diastolic LV pressure decay (Tau) and left ventricular end-diastolic pressure (LVEDP).

### Diastolic dysfunction grade, early relaxation and Tau

We proposed a Tau of greater than 48 ms as prolonged, [2, 21] with 45-56 ms [22–25] being two standard deviations above the mean in normal subjects. It would be expected that Tau would be prolonged in all grades of DD. However, impaired relaxation was present in only 49.0 % of patients with DD in this study. A prolonged Tau was also present in 38.8 % of patients without DD. These findings highlight the challenge of relying on echocardiographic grading of DD as an assessment of early relaxation. Other investigators have also shown no prolongation in Tau in patients with diastolic function abnormalities and elevated LVEDP [15].

At issue is the fact that no single echocardiographic parameter captures all the features of early relaxation. As early relaxation is impaired, IVRT increases, mitral annular movement is delayed and its velocity (e’) decreases, and mitral inflow changes, with a reduction in E wave height and lengthening of deceleration time. However, these parameters are all influenced by other factors. IVRT is dependent on the rate of relaxation, but also on the difference between aortic blood pressure and left atrial pressure. E wave height is affected by LVEDP, LV compliance, atrial conduit and booster pump function. Mitral annular e’ velocity is related to early relaxation, but is also heavily dependent on systolic function [26, 27].

### LV stiffness and LVEDP

LV stiffness and LVEDP are linked, as the diastolic rise in pressure is more pronounced in a less compliant ventricle. While there may be discrepancy between LVEDP and LV stiffness in the setting of acute changes of load, LVEDP would be expected to track LV stiffness in stable heart disease. Mitral inflow deceleration time (DT) has been shown to be inversely correlated with the operative stiffness of the LV (the change in ventricular pressure for a given change in volume) [28–30], and therefore with LVEDP. The ratio of E/e’ is also considered a measure of LVEDP [2, 31], and both of these parameters are incorporated into the current DD grading schema.

In our study, LVEDP was increased in comparison to those with normal diastolic function among grade 3 DD, but not grade 2 DD patients. Indeed, using a cutoff of 16 mmHg [17, 22, 24, 25], elevated LVEDP was only present in 28.4 % of subjects with DD. These findings are consistent with previous work showing that LV filling pressures [18, 19] and LV stiffness [32] are not always increased in patients with grade 2 DD. We used the additional measure of end diastolic volume index at 20 mmHg (EDVi20) as an estimate of LV compliance, and found that chamber compliance was actually *higher* (not lower as would be expected) in patients with more advanced DD.

Therefore, if grade 2 DD is meant to imply raised filling pressures beyond those seen in grade 1 DD and normal diastolic function, the accuracy of DD grading in describing filling pressures in this study was limited. Furthermore, grade 3 DD was associated with increased filling pressures and elevated BNP, but the ancillary measure of EDVi20 suggests that these patients do not as a rule have reduced ventricular compliance. Multivariate analysis suggests that the relationship between EDVi20 and DD grade may actually be driven by differences in LV systolic function.

### Consistency and application of diastolic function grading

Only 76 out of 405 patients with diastolic dysfunction (18.8 %) had unambiguous DD grading. We elected to assign patients to the DD category for which they had the greatest number of parameters, but this issue is not addressed in current guidelines [2]. Another important issue is the age-dependency of many of the parameters used to assign DD grade. Current guidelines do not propose age-specific cut-offs for any of these measures. This may lead to significant disagreement grading diastolic function in elderly individuals, and to some normal individuals being misclassified as having diastolic dysfunction.

On the other hand, findings that could be expected in patients with diastolic impairment are not universally seen by echocardiography. One important example would be left atrial enlargement. In our study, left atrial volume index (LAVi) was increased in patients with grade 3 but not grades 1 or 2 DD. This is in keeping with with the unexpectedly low LVEDP in patients with grade 2 DD in our cohort. It is also consistent with prior studies showing normal LAVi [19] or left atrial dimensions [18] among patients with what was felt to be significant diastolic dysfunction. Nevertheless, this led us to repeat our main analyses after excluding patients with DD who had LAVi <34 mL/m2. The findings were similar to those reported elsewhere in the manuscript. These data raise the question of whether or not there are a significant number of patients in practice who are falsely ascribed ‘normal’ diastolic function because of normal left atrial size.

### Importance of systolic function

We elected to study patients with both preserved and reduced ejection fraction. The diastolic function grading system in the current guidelines does not make a distinction between these two groups. Since they do have separate algorithms for estimating filling pressure in normal and depressed ejection fraction [2], there is precedence for this, and we think the differences between these groups are worth exploring. In the present study, LV systolic dysfunction was more predictive of Tau than was DD grade, and once systolic dysfunction was incorporated into multivariate analysis, there was no influence of DD grade on Tau.

### The paradigm of diastolic dysfunction grading

The patients in this report belong to a population with a high prevalence (88.1 %) of DD. However, the finding of prolonged Tau > 48 ms in only 47.5 % of patients, suggests a lower than expected rate of impaired relaxation. Previous investigators have also failed to show significant prolongation of Tau in patients with other markers of diastolic dysfunction [15]. While grade 1 patients were found to have prolonged Tau, this was not seen in grade 2 or grade 3 patients. Given the number of patients with abnormally elevated LVEDP but normal Tau, this begs the question of whether patients with increased operative stiffness of this kind should really be referred to as having ‘diastolic dysfunction’. If early relaxation is not impaired, some of these patients may have raised filling pressures simply because they are functioning at extreme levels of elevated preload. This notion is supported by our failure to show the expected reduction in EDVi20 at higher grades of DD.

Additionally, LVEDP was not found to be increased in grade 2 patients. Failure to show a clear stepwise increase in LVEDP with progressive grades of DD beyond grade 1 is consistent with previous reports showing no difference between resting filling pressures of patients with diastolic heart failure and those of control subjects [18, 19].

The findings of this study question the notion that DD is a predictable, progressive process beginning with impaired relaxation followed by reduced compliance and increased filling pressures. The prognostic value of echo graded DD may relate more to its reflection of intrinsic properties of the left ventricle, or to exercise hemodynamics [19] than to its correlation with resting hemodynamics alone. This would be supported by data from others showing the restrictive filling pattern of heart failure can be distinguished from volume overload in the normal heart [33].

### Limitations of the current study

An important technical limitation of this study is that echo images and catheterization tracings were not obtained simultaneously. This could lead to significant underestimation of the relationship beween invasive hemodynamics and echo parameters. However, echocardiographic DD is frequently reported and used clinically, with an expectation that it has diagnostic and prognostic value well outside of a 24 h timeframe. We used the electronic medical record to exclude patients in whom the use of diuretics, the provision of intravenous fluids, or a change in vasodilator therapy could have influenced hemodynamics.

We were only able to use a semi-invasive estimate of Tau because high fidelity pressure measurements were not made at the time of catheterization. Nevertheless, the assumptions used have been validated [20], assuming that LVEDP can be substituted for the left atrial pressure at the time of mitral valve opening. Similarly, we were not able to make measurements of static stiffness but relied on LVEDP and calculated EDVi20 as markers of stiffness/compliance.

A large percentage of cases were excluded, resulting in a fairly select group of patients in the final analysis. This was done to ensure that conditions affecting LV filling other than diastolic function were excluded. We feel that this is critical when evaluating DD, as supported by the guidelines [2].

Even with careful reanalysis of primary data, there is the possibility of misclassification of DD grade based on the echocardiographic data [14]. We attempted to reduce this by including only studies with complete Doppler data and high image quality. We also repeated our analyses after excluding patients who fulfilled criteria for DD based on low e’ values but not based on left atrial volumes, with similar results.

## Conclusions

Although echocardiographic grading of diastolic function was significantly related to invasive hemodynamics in this population, the relationship was modest. Impaired relaxation was not a universal finding in DD, not significantly differentiating patients with normal diastolic function by echocardiography from patients with grade 2 or 3 DD. No influence of DD grade on early relaxation was found after controlling for systolic dysfunction. Additionally, abnormally increased filling pressures were frequently seen among patients with normal invasive measures of early relaxation, questioning the current paradigm of diastolic function progression.
